# Preclinical efficacy investigation of human neutrophil elastase inhibitor sivelestat in animal model of psoriasis

**DOI:** 10.1002/ski2.90

**Published:** 2021-12-30

**Authors:** A. S. Zhukov, V. R. Khairutdinov, A. V. Samtsov, M. Krasavin, A. V. Garabadzhiu

**Affiliations:** ^1^ Department of Dermatology S. M. Kirov Military Medical Academy Saint Petersburg Russia; ^2^ Saint Petersburg State University Saint Petersburg Russia; ^3^ Saint Petersburg State Technological Institute (Technical University) Saint Petersburg Russia

## Abstract

**Background:**

Psoriasis is a chronic immune‐mediated inflammatory skin disease manifested by an increased rate of keratinocyte division. Currently, it has been established that the cytokines of the IL‐36 family play a significant role in the initiation and regulation of the inflammatory process in psoriasis. The IL‐36 cytokine found in skin is inactive and its activation requires proteolytic processing that may occur via the involvement of neutrophil serine proteases such as human neutrophil elastase (HNE). The localization of these enzymes in the upper layers of the epidermis suggests that topical application of HNE inhibitors could be efficacious in the treatment of psoriasis. Sivelestat is an HNE inhibitor developed for systemic use towards the treatment of acute respiratory failure.

**Aim:**

The present study focussed on the investigation of the effects of sivelestat formulated for topical use, in the imiquimod‐induced model of psoriasis in mice.

**Methods:**

The psoriasis‐like state was induced by application of imiquimod (Aldara^®^) 5% cream to mouse shaven skin. A group of 40 inbred mice of the BALB/c strain randomized into 4 groups of 10 was used in the experiment: Group 1 – no therapy (control), Group 2 – ointment (Vaseline) containing 1% sivelestat, Group 3 – cream (lanoline + olive oil + water in equal proportions) containing 1% sivelestat, Group 4 – 1% betamethasone dipropionate. Dermatological assessment of skin lesions was performed by means of the PASI method (mPASI), as well as histological and immunohistochemical evaluation.

**Results:**

Based on the evaluation of efficacy manifestations, it was established that the total mPASI index value decreased by 50% during therapy with sivelestat cream and by 36% during therapy with sivelestat ointment. Histological study revealed that the epidermal thickness in groups that underwent therapy was 2.4–3.6 times lower compared to the control group. Immunohistochemical study of the skin indicated that following sivelestat treatment, the quantity of CD3+cells in the skin was 1.8–2.2 times lower, and the level of proliferative activity (Ki‐67+cells) was 2.3–2.9 lower compared to the non‐therapy group. In contrast to topical corticosteroids where the more pronounced anti‐inflammatory effect is typically seen with ointment formulations, with sivelestat we observed an opposite effect. The reasons for that reversal remain unclear.

**Conclusion:**

Based on the results obtained using the animal model of imiquimod‐induced psoriasis, it was established that the HNE inhibitor sivelestat demonstrated efficacy comparable to that of a strong topical glucocorticoid steroidal drug (betamethasone dipropionate 1%). Significant resolution of skin lesions, reduction of epidermal thickness, diminishing of the skin infiltration with T‐lymphocytes and normalization of the cell division rate in epidermis and dermis were evident. Thus, suppression of IL‐36 mediated inflammation activity in the skin by topical application of a HNE inhibitor represents a promising new direction in the treatment of psoriasis. Certainly, HNE has other targets; thus, molecular studies could be subject of future experiments beyond the scope of the present study.

## Graphical abstract

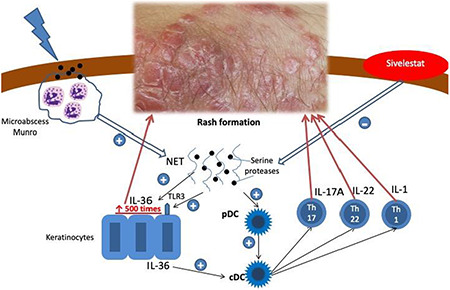

1

**What is already known about this topic?**

- One of the key cytokines in the pathogenesis of psoriasis is IL‐36 which requires poteolytic processing by human neutrophil elastase (HNE) for activation.
- HNE inhibitors have been preliminary shown to be efficacious in an animal model of psoriasis.

**What does this study add?**

- HNE inhibitor sivelestat approved for clinical use against acute respiratory failure produces efficacy results comparable to those of a a strong topical glucocorticoid steroidal drug.
- This data suggest an expedited path for repositioning of sivelestat in an entirely new therapeutic area as a first‐in‐class treatment for psoriasis.

## 2 INTRODUCTION

Psoriasis is a chronic multifactorial immune‐mediated inflammatory skin disease characterized by an increased rate of keratinocyte division. One of the key cytokines in the pathogenesis of psoriasis is IL‐36. Genetic variations of this cytokine receptor are associated with the development of pustular forms of the disease.^1^ Recent data also suggest a major role of IL‐36 in the pathogenesis of plaque psoriasis.^2^, ^3^

Analysis of the literature data as well as our own studies allowed establishing that cytokines of IL‐36 family (α, β, γ) are overexpressed in the skin of psoriatic patients.^4^, ^5^ The association of IL‐36γ level with the severity of the disease was established as well as the significance of this marker in differential diagnosis with other chronic dermatoses.^5^

Studies involving an animal model of psoriasis revealed the key role of IL‐36 in the development of inflammatory reaction. It was determined that genetically modified mice overexpressing IL‐36α develop a psoriatic phenotype, that is thickening of the skin, erythema and peeling. At the same time, blockage of the IL‐36 receptor (IL‐36R) led to the disappearance of the lesions.^4^

**FIGURE 1:**
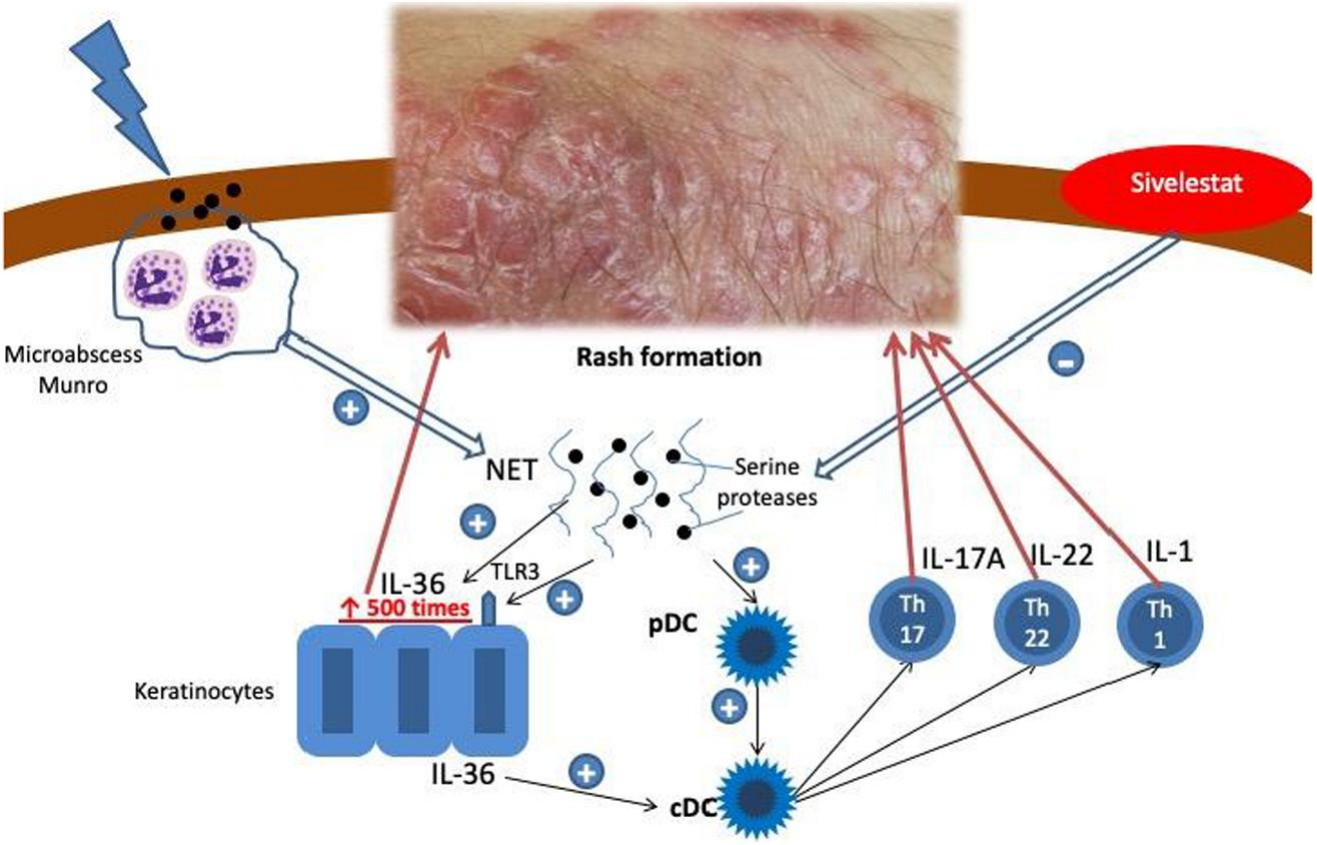
Formation of the IL‐36 mediated inflammatory response in patients with psoriasis. cDC, myleoid dendritic cells; IL‐1, 17A, 22, 36‐interleukins 1, 17А, 22, 36; NET, neutrophil extracellular traps; pDC, plasmacytoid dendritic cells; TLR, Toll‐like receptors; Th17, Th22, Th1, Type I Т‐cells 17‐, 22‐

IL‐36 cytokine is expressed in the skin in an inactive form. Its activation requires proteolytic processing which may involve neutrophil serine proteases (such as neutrophil elastase, cathepsin G, proteinase‐3) contained in neutrophil granulocytes (Figure 1).

Development of the Munro microabscesses, consisting of neutrophils infiltrating from the papillary dermis into the epidermal stratum corneum, is one of the key histological symptoms of psoriasis.^6^ Having entered the epidermis through the blood flow, neutrophils release net‐like structures that contain serine proteases. The latter are key players in the proteolytic processing of IL‐36, thereby increasing its activity by up to 500 times, which leads to the degradation of a range of structural proteins (elastin, proteoglycan, collagen and fibronectin) of the extracellular matrix.^7^ All these factors may contribute to the formation of the autoimmune response in psoriasis.

Overexpression of neutrophil serine proteases in the upper layers of the epidermis suggests the possibility of using topically administered inhibitors.^8^ This certainly carries obvious advantages from the standpoint of occurrence of adverse reactions to such drugs compared to systemic therapeutic agents. In the present study, we aimed to investigate a topical therapeutic agent that would suppress the activity of IL‐36 mediated psoriatic inflammation of the skin via inhibiting serine proteases, from the standpoint of its efficacy in an animal model of psoriasis. To this end, we selected human neutrophil elastase inhibitor, sivelestat (trade name Elaspol^®^) which was developed by Ono Pharmaceutical Co.^9^ for systemic therapy of acute respiratory distress syndrome where the level of neutrophil proteases plays a central role in the pathogenesis.^10^ Herein, we report on the results of this study.

## 3 MATERIALS AND METHODS

To prepare sivelestat cream, 1% powered sivelestat was dissolved in distilled water containing 5% DMSO, until full dissolution was observed at 40°C. Then olive oil and lanoline were added in equal volumes whereupon the cream was stirred until a fine suspension was observed. The sivelestat ointment was prepared by dissolving powdered sivelestat in a minimum amount of DMSO and mixing is with such an amount of Vaseline at 40°C so that the DMSO concentration did not exceed 5%, until fine‐consistency ointment was obtained. Both the cream and the ointment were stored at 4°C and used within 5 days after being brought to room temperature.

**FIGURE 2:**
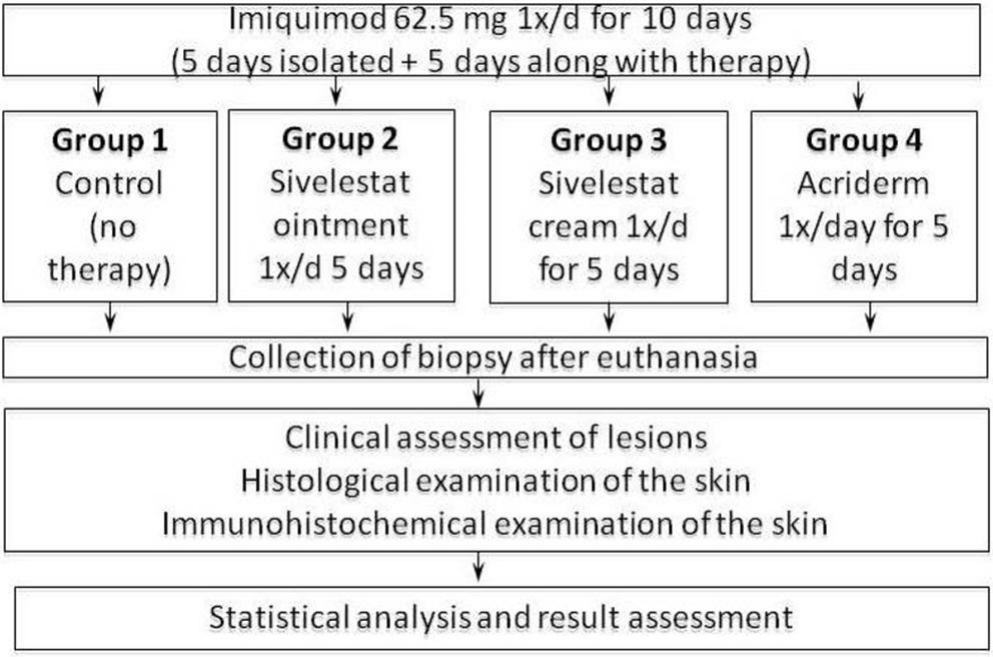
Study workflow

The experiment used a group of 40 inbred mice of the BALB/c strain randomized into 4 groups of 10. The formation of psoriasis‐like inflammation of the skin was induced by daily application of imiquimod (Aldara) to the shaved skin on the backs of mice, once a day for 10 (Figure 2).^11^

Mice in Group 1 did not undergo therapy (control). Starting on the sixth day of the model formation, 1 h after applying imiquimod, Group 2 mice had the 1% sivelestat ointment (Vaseline) applied 1x/day for 5 days, Group 3 mice—cream (lanoline + olive oil + water in equal amounts) containing 1% Sivelestat 1x/day for 5 days, and Group 4 mice—betamethasone dipropionate 1% (Acriderm) 1x/day for 5 days. Daily clinical assessment of skin lesions was performed by the PASI method (mPASI). It determined the following parameters: erythema, induration, peeling of the skin on a 5‐point scale from 0 to 4. On the 11th day of the experiment all mice were euthanized and biopsied. Histological (assessment of the thickness of the epidermis, expression of acanthosis, hyperkeratosis, and inflammatory infiltrate) and immunohistochemical (determination of the proliferative activity of epidermal cells–Ki 67 marker, presence of lymphocytes–CD3 marker) examinations were carried out on the obtained bioptats. The expression of acanthosis, hyperkeratosis and inflammatory infiltrate was evaluated on a 4‐point scale («−» – absent expression, «+» – weak expression, «++» – moderate expression, «+++» – strong expression). The number of labelled cells was calculated at a magnification of 200x on a field with an area of 0.34 mm^2^.

Statistical data processing was carried out using the programme STATISTICA 10.0 (StatSoft, Inc). The Mann–Whitney U‐test was used to compare the data between the groups. Differences were considered significant at p < 0.05.

## 4 RESULTS

**FIGURE 3:**
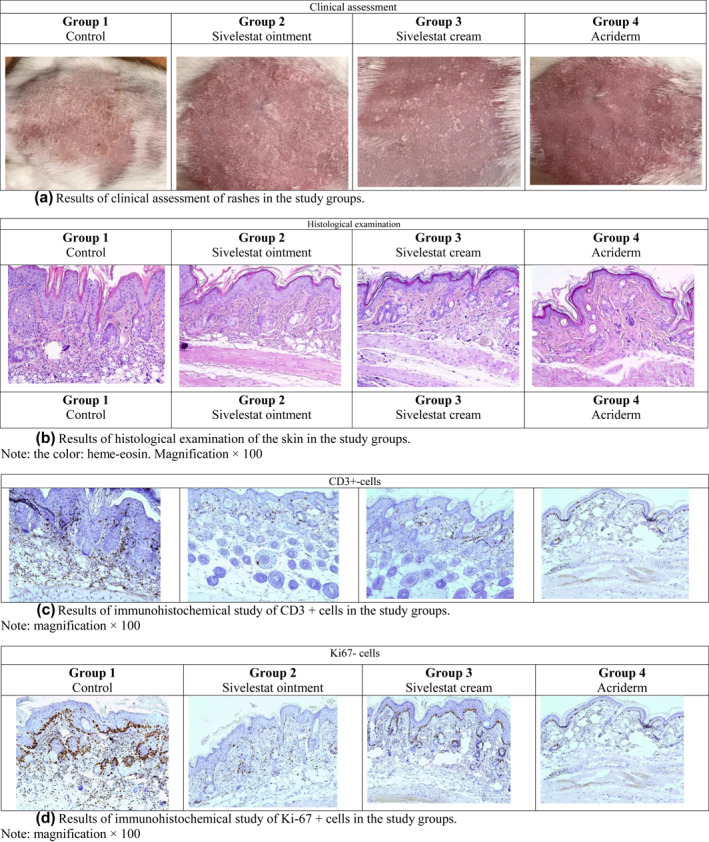
(a) Results of clinical assessment of the rash in the study groups; (b) Results of histological examination of the skin in the study groups (stain: haem‐eosin, magnification × 100); (c) Results of immunohistochemical study of CD3+cells in the study groups (magnification × 100); (d) Results of immunohistochemical study of Ki‐67+cells in the study groups (magnification × 100)

During the induction of the psoriasis model on day 5, a psoriasiform phenotype was formed in laboratory animals: erythema, peeling, thickening of the skin. On day 10 of the experiment in Group 1 (nontherapy), clinical changes were strongly expressed (Figure 3).

When comparing the results of the mPASI assessment, it was found that the value of this indicator in the groups of animals receiving sivelestat cream/ointment and Acriderm was significantly lower than in the group without treatment (p < 0.05). The lowest values were observed in the group receiving a glucocorticosteroid drug (a decrease in mPASI by 83%) and in the group where sivelestat cream was used (a decrease in mPASI by 50%) (Table 1).

**TABLE 1:** Clinical assessment of the severity of skin changes in animals in the study groups on the 10th day of the experiment

Entry	Group	Erythema	Induration	Peeling	mPASI
1	Control	2.5 [1.9–2.8]	2.9 [2.1–3.2]	2.7 [2.2–3.1]	8.1 [7.4–8.7]
2	Sivelestat ointment	2.1 [0.5–1.3]	2.4 [0.6–1.2]	0.7 [0.7–1.2]	5.2 [2.2–3.2]*
3	Sivelestat cream	1.5 [0.9–1.9]	1.6 [1.0–2.1]	0.9 [0.4–1.2]	4.0 [0.7–1.4]*
4	Acriderm	0.4 [0.1–0.7]	0.7 [0.2–1.0]	0.1 [0.0–0.2]	1.3 [1.0–1.8]*

*Note*: The data is presented as M [IQR], where M is the median, IQR is the interquartile interval.

*p < 0.05 when compared with the control group.

When assessing histological changes in Group 1 (non‐therapy), characteristic signs of psoriasis were revealed: in the epidermis–expressed acanthosis and parakeratosis, in the dermis–lymphohistiocytic infiltrate and dilated vessels (Figure 3). In Group 4 (Acriderm), pathomorphological changes were practically absent. In Groups 2 (sivelestat ointment) and 3 (sivelestat cream), no expressed histopathological changes in the skin were detected: moderate acanthosis, focal hyperkeratosis, and scanty lymphohistiocytic infiltrate around the slightly dilated vessels of the papillary dermis (Table 2). It was established that the thickness of the epidermis in animals of Group 1 (non‐therapy) was 2.4–3.6 times greater than in the groups where the therapy was used (p < 0.05). Pairwise comparison of groups receiving different therapies revealed no significant differences in the thickness of the epidermis.

**TABLE 2:** Histological changes in the skin of animals in the study groups on the 10th day of the experiment

Entry	Group	Epidermal thickness, μm	Acanthosis	Hyperkeratosis	Lymphohistocytic infiltrate
1	Control	97 [84–98]	+++	+++	+++
2	Sivelestat ointment	40 [37–47]*	++	++	+
3	Sivelestat cream	32 [31–44]*	+	+	+
4	Acriderm	27 [23–29]*	‐	+	‐

*Note*: The data is presented as M [IQR], where M is the median, IQR is the interquartile interval.

*p < 0.05 when compared with the control group.

Immunohistochemical examination of the skin revealed a high content of CD3+cells in the control group. After treatment with the drug sivelestat the number of CD3^+^ cells in the skin was 1.8–2.2 times lower than in the group without treatment (p < 0.05).

Assessment of the level of proliferative activity (Ki‐67+cells) of the skin showed that in the groups that used the drug sivelestat this figure is 2.3–2.9 times lower than in the group without treatment (p < 0.05). The betamethasone dipropionate group had the lowest levels of CD3 and Ki67 expression compared to the control group and the sivelestat cream/ointment groups (p < 0.05 in pairwise comparisons) (Table 3).

**TABLE 3:** The level of expression of immunohistochemical markers of animal skin in the study groups on the 10th day of the experiment

Entry	Group	CD3+‐cells	Ki67+‐cells
1	Control	101 [79–135]	316 [292–334]
2	Sivelestat ointment	54 [48–55]*	109 [105–115]*
3	Sivelestat cream	45 [43–52]*	140 [81–143]*
4	Acriderm	7 [6–11]*	52 [50–56]*

*Note*: The data is presented as M [IQR], where M is the median, IQR is the interquartile interval.

*p < 0.05 when compared with the control group.

## 5 DISCUSSION

When choosing external remedies, the arsenal of a dermatovenerologist includes several groups of drugs: glucocorticosteroids containing a synthetic analogue of vitamin D, salicylic acid, tar and some others. Their main disadvantage is the non‐specific effect on the key links in the development of the inflammatory process in psoriasis, which can manifest itself as the emergence of adverse events and insufficient activity.

Drugs that inhibit TNFa, IL‐17A and IL‐23 have shown the greatest clinical effectiveness in the treatment of patients with psoriasis, but the systemic immunosuppressive effect, the need for regular administration and the high cost limit their use.

The principal difference in the realization of the effects of IL‐36 from other pro‐inflammatory cytokines is its activation in the skin directly in the focus of inflammation with the participation of serine proteases (Figure 1). In addition, the production of IL‐36 during trauma allows us to explain the formation of the Koebner phenomenon and the characteristic localization of lesions. When the skin is damaged, Toll‐like type 3 receptors (TLR3) are activated on keratinocytes, which leads to the release of IL‐36.^12^ Activated IL‐36 induces the synthesis of IL‐23 by dendritic cells, triggering IL‐17‐mediated inflammation on the one hand, and initiates the synthesis of antimicrobial regenerative proteins (REG3A) and vascular endothelial growth factor A (VEGF‐A) in keratinocytes—on the other. Activation of these factors promotes keratinocyte proliferation, epidermal hyperplasia, and endothelial cell proliferation, leading to neoangiogenesis.^12^

IL‐36 in the skin is mainly synthesized by keratinocytes, in contrast to IL‐17A and IL‐23, which are expressed mainly by immunocompetent cells. Thus, local inhibition of IL‐36 is expected to significantly reduce the effect on systemic immune processes and improve the safety profile.

In this work, for the first time, we conducted a study of a topical targeted drug‐serine proteases inhibitor, the therapeutic effect of which is aimed at suppressing the activity of IL‐36. The advantage of choosing this point of exposure is the ability to control the inflammatory process in psoriasis at the early stages of its development. It is noteworthy to highlight the high selectivity, the topical form of the drug, the relative cheapness and ease of production of the chemical compound.

In the work on the classic laboratory model of imiquimod‐induced psoriasis, a pronounced clinical improvement in the skin condition was shown when using the drug sivelestat, which was manifested by a decrease in the intensity of erythema, peeling and skin thickness. Histological examination revealed that the thickness of the epidermis up to 3 times lower. The severity of acanthosis, hyperkeratosis, and lymphohistiocytic infiltrate also abated. The objective criterion for the effectiveness of sivelestat is the result of an indirect immunohistochemical study. It was shown that the number of T‐lymphocytes (CD3+cells) in the skin during treatment with sivelestat decreased by a factor of 1.8–2.2, and the proliferative activity of epidermal cells (the number of Ki‐67+cells) by 2.3–2.9. The more pronounced clinical efficacy of the sivelestat cream compared to the ointment requires further study.

When comparing clinical and histological changes, no significant differences were found between the Acriderm and sivelestat cream groups. At the same time, the results of indirect immunohistochemical studies (CD3 and Ki67 markers) showed a more pronounced decrease in the level of T‐lifocytes and proliferative activity of skin cells in the group of topical glucocorticosteroid drugs, which can be explained by its strong vasoconstrictor effect.

The results of the study demonstrated the antiproliferative, anti‐inflammatory and resolving effects of the drug sivelestat, which allows us to recommend its further study for the treatment of patients with psoriasis.

## 6 CONCLUSION

In a laboratory model of imiquimod‐induced psoriasis, it was found that serine protease inhibitor sivelestat has a clinical efficacy comparable to that of a strong topical glucocorticosteroid drug (betamethasone dipropionate 1%). It shows a pronounced resolution of the elements of the skin lesions: erythema, induration and peeling, as well as their total indicator‐the mPASI index; a significant reduction in inflammatory changes in the skin (reduction in the thickness of the epidermis and the number of lymphohistiocytic infiltrates); reduction of skin infiltration by T‐lymphocytes and normalization of the rate of cell division of the epidermis and dermis.

Suppression of the activity of IL‐36‐mediated inflammation in the skin by topical serine protease inhibitors is a new promising direction in the treatment of patients with psoriasis. This will allow us to improve approaches to the treatment of both limited and common forms of the disease.

## CONFLICT OF INTEREST

None to declare.

## AUTHOR CONTRIBUTIONS

Alexander Zhukov: Investigation; Equal, Methodology; Equal, Project administration; Equal, Validation; Equal, Visualization; Equal, Mikhail Krasavin: Conceptualization; Equal, Methodology; Equal, Writing – original draft; Equal, Writing – review & editing; Equal, Alexey Samtsov: Conceptualization; Equal, Vladislav R. Khairutdinov: Conceptualization; Equal, Funding acquisition; Equal, Project administration; Equal, Supervision; Equal, Writing – original draft; Equal, Alexander Garabadzhiu: Conceptualization; Equal, Funding acquisition; Equal, Project administration; Equal, Writing – review & editing; Equal.

## ETHICS STATEMENT

The study protocol was approved by the Independent Ethics Committee of the S. M. Kirov Military Medical Academy, Saint Petersburg.

## Data Availability

The data that support the findings of this study are available from the corresponding author upon reasonable request.
